# Diagnostic spectrum of unplanned acute hospital admissions within 30 days after emergency department presentation for vertigo in older adults: a province-wide retrospective cohort study

**DOI:** 10.3389/fmed.2026.1862951

**Published:** 2026-08-31

**Authors:** İskender Aksoy, Mehmet Ekiz

**Affiliations:** Department of Emergency Medicine, Faculty of Medicine, Giresun University, Giresun, Türkiye

**Keywords:** aged, dizziness, emergency service, hospitalization, stroke, vertigo

## Abstract

**Background:**

Vertigo is a common reason for emergency department (ED) attendance and is frequently evaluated with a primary focus on excluding stroke. However, the broader spectrum of serious diagnoses following vertigo presentation in older adults remains insufficiently characterized. We aimed to characterize 30-day hospital admissions among patients aged 65 years and older presenting to the ED with vertigo, with particular attention to diagnoses beyond stroke.

**Methods:**

This province-wide retrospective cohort study included patients aged ≥65 years presenting to 17 EDs in Giresun, Türkiye, with vertigo-related ICD-10 codes between January 2024 and December 2025. ICD-10 coding was used as an initial case-finding strategy, and only patients in whom vertigo was confirmed as the dominant presenting complaint after clinical record review were included in the final analysis. Patients were categorized according to admission timing; clinical characteristics, imaging use, risk scores, and final diagnoses were summarized descriptively, with exploratory between-group comparisons.

**Results:**

Among 6,460 older adults identified through vertigo-related ICD-10 codes, 269 had an unplanned acute hospital admission through an ED within 30 days after the index visit. After clinical record review, 186 patients with vertigo as the dominant presenting complaint were included in the final analysis. The mean age of the study cohort was 75.9 ± 7.2 years, and 106 patients (57.0%) were male. Seventy patients (37.6%) were admitted during the index ED visit, whereas 116 (62.4%) were admitted after ED discharge. Neurological emergencies accounted for 84 admissions (45.2%), of which 74 (39.8% of the total cohort) were strokes. Among the 116 admissions occurring after ED discharge, 79 (68.1%) were attributable to diagnoses other than stroke, including 71 (61.2%) due to non-neurological conditions, most commonly metabolic/renal, infectious, and cardiac disorders.

**Conclusion:**

Among older adults admitted within 30 days after an ED presentation with vertigo, non-neurological diagnoses constituted a substantial proportion of hospitalizations after ED discharge. These findings describe the diagnostic spectrum of a hospitalization-conditioned cohort and should not be interpreted as estimates of overall admission risk or diagnostic accuracy.

## 1 Introduction

Dizziness is an umbrella term encompassing several distinct symptom experiences, including vertigo, presyncope, disequilibrium, and nonspecific light-headedness. Vertigo is conceptually narrower and refers specifically to an illusion of self-motion or environmental motion. Although many vertigo presentations are attributable to benign peripheral vestibular disorders, the possibility of central pathology, particularly stroke, has driven extensive research and shaped contemporary emergency department (ED) practice. Older adults represent a particularly vulnerable group because both vertigo and broader dizziness presentations may occur in the context of multimorbidity, atypical clinical manifestations, and serious underlying disease. In older adults, vertigo may reflect not only vestibular disorders and central neurological events, but also non-neurological systemic illness. Large cohort analyses have shown that a small but clinically relevant proportion of patients discharged with dizziness are subsequently diagnosed with stroke or experience adverse neurological outcomes (1–3). In patients presenting with acute vestibular syndrome, central causes including posterior circulation stroke remain a critical diagnostic concern (4). In the present manuscript, the term “dizziness” is used when referring to the broader symptom literature or to populations defined as such in previous studies, whereas “vertigo” is used for the clinically confirmed symptom defining the present study cohort.

To address this risk, structured bedside examination strategies and clinical decision tools have been developed. Algorithms based on timing and triggers have been proposed to improve diagnostic accuracy in acute dizziness (5), and bedside approaches such as HINTS have demonstrated high sensitivity for central causes when performed by trained clinicians (6, 7). In parallel, clinical risk scores including TriAGe+ and the Sudbury Vertigo Risk Score aim to stratify the probability of serious underlying pathology (8, 9). External validation studies have reported moderate performance for identifying central neurological causes (10, 11). However, these tools were primarily designed to detect strokes or other central disorders.

In geriatric emergency medicine, the concept of “non-specific complaints” recognizes that subtle symptoms such as dizziness may conceal significant underlying illness (12). Metabolic disturbances including hyponatremia (13, 14), acute kidney injury (15, 16), and cardiac conduction abnormalities such as bradycardia (17) may initially manifest with dizziness or imbalance rather than overt organ-specific findings. Infectious processes in older adults may also present atypically, further complicating early recognition (18, 19).

Despite the breadth of potential diagnoses, most prior studies evaluating ED vertigo have focused predominantly on stroke incidence as the principal outcome. Data describing the full spectrum of serious diagnoses following vertigo presentation in older adults remain limited. Understanding this broader clinical distribution is essential in ageing populations with high comorbidity burden.

The aim of this study was to characterize the diagnostic spectrum of hospital admissions occurring within 30 days among patients aged 65 years and older presenting to the ED with vertigo as the dominant symptom, with particular attention to diagnoses beyond stroke. By focusing on a clinically confirmed vertigo cohort of older adults, we sought to describe the serious diagnoses associated with hospital admissions occurring within the predefined 30-day observation period after the index ED presentation. These subsequent diagnoses were evaluated as final hospital diagnoses and were not presumed to represent etiological classifications of vertigo. We also aimed to examine whether admissions after ED discharge were frequently attributable to non-neurological systemic conditions rather than cerebrovascular events alone.

## 2 Materials and methods

### 2.1 Study design and setting

This province-wide retrospective observational cohort study was conducted in Giresun, Türkiye, and included all seventeen hospitals in the province with active emergency department (ED) services. Emergency department presentations between 1 January 2024 and 31 December 2025 were screened. The study was designed to characterize the diagnostic spectrum of hospital admissions occurring within 30 days among older adults presenting to the ED with vertigo as the dominant symptom, rather than to estimate the overall incidence of all subsequent outcomes in the full vertigo population.

### 2.2 Data source and case identification

Data were obtained from the hospital information management system. ED visits were screened using ICD-10 codes commonly applied to vertigo-related presentations, including R42 (dizziness and giddiness), H81.1 (benign paroxysmal vertigo), H81.3 (other peripheral vertigo), and H81.4 (vertigo of central origin). In routine ED practice, most vertigo-related presentations were coded as R42.

Because ICD-10 code R42 encompasses symptoms broader than vertigo, including nonspecific dizziness, light-headedness, faintness, and presyncope, ICD coding was used solely as an initial case-finding strategy. Final inclusion required explicit documentation of vertigo, defined clinically as an illusion of self-motion or environmental motion, as the primary presenting complaint and the main reason for ED evaluation. Isolated nonspecific dizziness, light-headedness, faintness, presyncope, imbalance, or gait unsteadiness without documented vertigo was not considered sufficient for inclusion.

Cases were excluded when vertigo was not the primary presenting complaint but was documented only as a secondary symptom or diagnosis associated with another dominant reason for ED attendance. The initial clinical-record screening was performed by one investigator using the predefined inclusion and exclusion criteria described above. Examples included visits for medication renewal, blood pressure measurement, trauma, syncope, predominant nausea/vomiting, or another acute complaint in which vertigo-related coding represented a secondary or administrative diagnosis. Cases considered potentially ambiguous during the initial review were subsequently discussed by the authors, and the final inclusion or exclusion decision was reached by consensus. Because the records were not independently classified by two blinded reviewers, formal inter-rater agreement was not calculated. Data extraction was performed after completion of the 30-day follow-up period to ensure complete ascertainment of hospital admissions.

### 2.3 Participants and definitions

The study population consisted of patients aged ≥65 years who presented to an included ED with a vertigo-related ICD-10 code and subsequently underwent an unplanned acute hospital admission through an ED within 30 days of the index visit. Elective admissions, planned hospitalizations, and admissions originating directly from outpatient clinics were not included. The age threshold of 65 years was selected *a priori* because the study specifically focused on older adults, a population in whom vertigo presentations are more likely to be associated with multimorbidity, atypical manifestations, and serious neurological or systemic disease.

The primary endpoint was an unplanned acute hospital admission through an ED within 30 days after the index ED visit. A 30-day follow-up window was selected *a priori* as a clinically relevant short-term period for identifying subsequent hospital admissions. However, inclusion within this time window was based on temporal proximity alone and does not establish that a later admission was causally related to the index ED presentation. As a supplementary safety outcome, 30-day all-cause mortality was assessed among all 6,460 older adults identified through vertigo-related ICD-10 codes. Mortality status and dates of death were obtained from the national Death Notification System. Deaths were categorized according to whether they occurred after hospital admission or without any recorded hospital admission during the 30-day follow-up period.

Patients were categorized according to the timing of admission: Group A included patients admitted during the index ED visit; Group B included patients admitted within 1–7 days after ED discharge; and Group C included patients admitted within 8–30 days after ED discharge. This grouping was intended to describe the timing of early and later admissions after ED discharge. Admission timing should not be interpreted as a direct measure of diagnostic error or as evidence that the condition responsible for the subsequent admission was present during, caused by, or otherwise related to the index ED presentation.

Because the study was based on presenting symptom complexes rather than uniform final vertigo subtype diagnoses, cases were not retrospectively classified across the entire cohort into a strict peripheral-vs.-central vertigo framework. Instead, final hospital discharge diagnoses were used to describe the spectrum of neurological and non-neurological conditions identified during follow-up. Cerebrovascular diagnoses were defined according to the final recorded hospital diagnosis and discharge documentation rather than retrospective reinterpretation by the authors.

### 2.4 Variables and data collection

Demographic variables included age and sex. Clinical data were extracted from structured electronic records, and no additional reinterpretation of physician notes was performed beyond confirmation of the presenting complaint. ED triage was recorded and analyzed as high priority (yellow/red) vs. low priority (green). Accompanying symptoms and clinical findings included nausea/vomiting, headache, gait disturbance/imbalance/ataxia, syncope/presyncope, acute focal neurological symptoms, visual symptoms, and chest pain or dyspnea.

Recorded comorbidities were hypertension, diabetes mellitus, atrial fibrillation, prior stroke or transient ischemic attack, coronary artery disease, chronic kidney disease, chronic obstructive pulmonary disease/asthma, and malignancy. Baseline medication use included antiplatelet therapy, anticoagulant or direct oral anticoagulant therapy, antihypertensive therapy, and antihyperlipidemic therapy.

Neuroimaging performed during the index ED visit was recorded as cranial computed tomography (CT) and/or brain diffusion magnetic resonance imaging (MRI). Imaging findings were classified according to the original radiology reports. Acute CT findings included radiologist-reported acute ischemic changes, acute infarction, or intracranial hemorrhage, whereas acute diffusion MRI findings were defined as restricted diffusion compatible with acute ischemic infarction. Chronic findings included previously established infarcts, chronic small-vessel ischemic changes, cerebral atrophy, and other non-acute abnormalities. Neuroimaging was not performed routinely in all patients but was obtained according to the clinical judgment of the treating emergency physician in routine practice. Likewise, because of the retrospective real-world design, no standardized prospective neurological examination protocol was applied across centers.

Two clinical risk scores, TriAGe+ and the Sudbury Vertigo Risk Score, were calculated retrospectively using routinely recorded vital signs, medical history, presenting symptoms, and physical examination findings in the hospital information management system. Complete documentation was available for all required components of both scores in all 186 patients; therefore, both scores were calculated for the entire study cohort. No undocumented component was assumed to be absent, negative, or zero, and no imputation was performed.

For hospitalized patients, the time from the index ED visit to admission, admission service, admission unit, length of hospital stay, and final discharge diagnosis were recorded. Final diagnoses were additionally grouped into broader categories as neurological, cardiovascular, infectious, metabolic/renal, pulmonary, and surgical emergencies.

### 2.5 Statistical analysis

Statistical analyses were performed using IBM SPSS Statistics for Windows, version 22.0 (IBM Corp., Armonk, NY, USA). Continuous variables were assessed for normality using the Shapiro–Wilk and Kolmogorov–Smirnov tests. Normally distributed variables were presented as mean ± standard deviation and compared using the independent samples t-test or one-way analysis of variance, as appropriate. Non-normally distributed variables were presented as median (interquartile range) and compared using the Mann–Whitney *U*-test or Kruskal–Wallis test, as appropriate. Categorical variables were summarized as number (percentage). Pearson's chi-square test was used when its assumptions were satisfied, Fisher's exact test was used for 2 × 2 tables with small expected cell counts, and the Fisher–Freeman–Halton exact test was used for larger r × c contingency tables. For the overall 6 × 3 comparison of the major diagnostic categories presented in Table 2, the Fisher–Freeman–Halton test was performed using Monte Carlo estimation based on 100,000 sampled tables. Pairwise comparisons following statistically significant omnibus tests were adjusted using the Bonferroni method. No global adjustment was applied for the multiplicity of comparisons across variables and tables; therefore, all *p* values should be interpreted as exploratory and hypothesis-generating rather than confirmatory. Because of the small subgroup sizes, the analyses presented in Table 4 were descriptive only, and no formal between-group hypothesis testing was performed. A two-sided *p* value < 0.05 was considered statistically significant.

### 2.6 Ethics

The study protocol was approved by the Giresun Training and Research Hospital Scientific Research Ethics Committee (BAEK-584; decision no: 10; approval date: 4 February 2026). Because of the retrospective design and the use of routinely collected anonymized data, the requirement for individual informed consent was waived by the ethics committee. All procedures were conducted in accordance with the principles of the Declaration of Helsinki.

## 3 Results

### 3.1 Study cohort and admission timing

**Figure 1:**
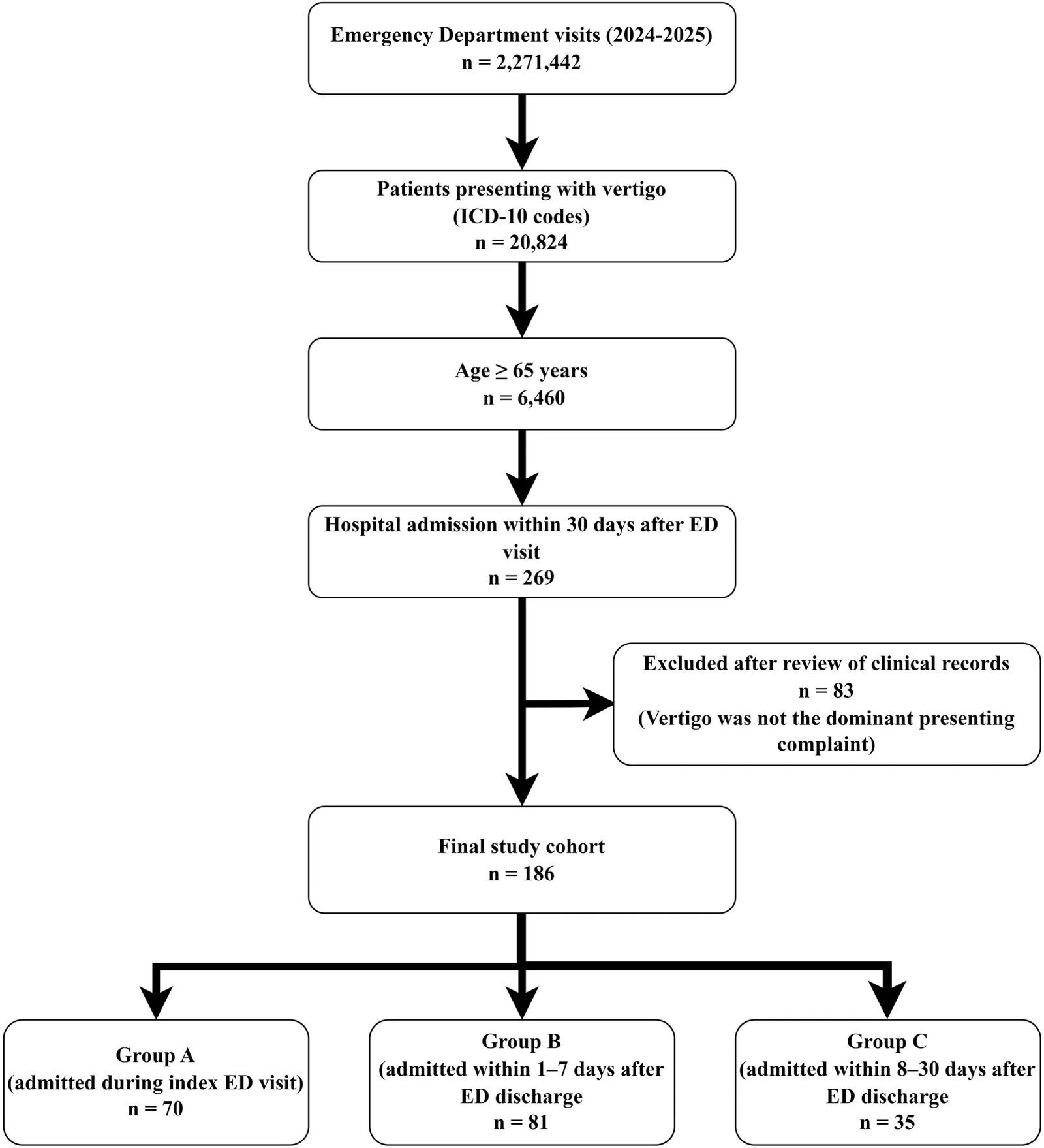
Flow diagram of patient selection and classification.

During the study period, 2,271,442 patients presented to the ED. Among these visits, 20,824 were identified through vertigo-related ICD-10 codes, including 6,460 patients aged ≥65 years. Within 30 days of the index ED visit, 269 of these older patients had hospital admission records. After clinical record review, 83 patients were excluded because vertigo was not the dominant presenting complaint, and the remaining 186 patients constituted the final study cohort (Figure 1). Patients were classified according to the timing of admission as Group A (admitted during the index ED visit, *n* = 70), Group B (admitted within 1–7 days after discharge, *n* = 81), and Group C (admitted within 8–30 days after discharge, *n* = 35). Thus, vertigo-coded presentations accounted for 0.9% of all ED visits during the study period, and older adults represented 31.0% of these presentations.

### 3.2 Baseline clinical characteristics according to admission timing

The baseline clinical characteristics of the study population are presented in Table 1. Baseline characteristics were largely comparable across groups in terms of age, sex, comorbidities, medication use, and length of hospital stay. Exploratory comparisons showed variation across the admission-timing groups in triage category, acute focal neurological symptoms, and neuroimaging use (Table 1). Because triage acuity and imaging decisions are closely related to presenting clinical features, physician decision-making, and the subsequent admission pathway, these findings are presented descriptively and should not be interpreted as independent predictors or measures of diagnostic performance.

**Table 1:** Baseline demographic and clinical characteristics of patients admitted within 30 days of ED presentation for vertigo.

Variable	Total	Group A (*n* = 70)	Group B (*n* = 81)	Group C (*n* = 35)	*p*
Age (years)	75.9 ± 7.2	76.4 ± 7.4	75.6 ± 7.2	75.5 ± 6.8	0.755
Gender					
Female	80 (43.0)	31 (44.3)	38 (46.9)	11 (31.4)	0.292
Male	106 (57.0)	39 (55.7)	43 (53.1)	24 (68.6)	
Triage category					
Low-priority	85 (45.7)	0 (0)	58 (71.6)	27 (77.1)	<0.001
High-priority	101 (54.3)	70 (100)	23 (28.4)	8 (22.9)	
Nausea/Vomiting	76 (40.9)	31 (44.3)	37 (45.7)	8 (22.9)	0.055
Headache	13 (7.0)	1 (1.4)	8 (9.9)	4 (11.4)	0.066
Gait disturbance/imbalance	42 (22.6)	20 (28.6)	17 (21.0)	5 (14.3)	0.231
Syncope/Presyncope	20 (10.8)	11 (15.7)	7 (8.6)	2 (5.7)	0.213
Acute focal neurological symptoms	18 (9.7)	13 (18.6)	5 (6.2)	0 (0)	0.004
Visual symptoms	2 (1.1)	1 (1.4)	1 (1.2)	0 (0)	0.786
Chest pain or dyspnea	13 (7.0)	4 (5.7)	5 (6.2)	4 (11.4)	0.517
Time to admission (days)	1 (0–4)	0 (0–0)	1 (1–3)	16 (12–25)	
Admission unit					
Ward	150 (80.6)	58 (82.9)	65 (80.2)	27 (77.1)	0.778
Intensive care unit	36 (19.4)	12 (17.1)	16 (19.8)	8 (22.9)	
Length of hospital stay (days)	6 (4–11)	6 (4–9)	6 (4–10.5)	6 (4–15)	0.785
Hypertension	129 (69.4)	48 (68.6)	53 (65.4)	28 (80.0)	0.290
Diabetes mellitus	63 (33.9)	24 (34.3)	24 (29.6)	15 (42.9)	0.383
Atrial fibrillation	26 (14)	8 (11.4)	11 (13.6)	7 (20.0)	0.486
Previous stroke or TIA	21 (11.3)	9 (12.9)	6 (7.4)	6 (17.1)	0.274
Coronary artery disease	50 (26.9)	15 (21.4)	23 (28.4)	12 (34.3)	0.345
Chronic kidney disease	8 (4.3)	1 (1.4)	3 (3.7)	4 (11.4)	0.055
COPD or asthma	27 (14.5)	11 (15.7)	12 (14.8)	4 (11.4)	0.837
Malignancy	11 (5.9)	4 (5.7)	6 (7.4)	1 (2.9)	0.632
Cranial CT performed	107 (57.5)	54 (77.1)	45 (55.6)	8 (22.9)	<0.001
Normal	87 (81.3)	40 (74.1)	40 (88.9)	7 (87.5)	0.154
Acute findings	20 (18.7)	14 (25.9)	5 (11.1)	1 (12.5)	
Brain diffusion MRI performed	91 (48.9)	51 (72.9)	34 (42.0)	6 (17.1)	<0.001
Normal	47 (51.6)	15 (29.4)	26 (76.5)	6 (100)	<0.001
Acute findings	44 (48.4)	36 (70.6)	8 (23.5)	0 (0)	
Antiplatelet therapy	72 (38.7)	26 (37.1)	31 (38.3)	15 (42.9)	0.847
Anticoagulant therapy	21 (11.3)	7 (10.0)	8 (9.9)	6 (17.1)	0.478
Antiplatelet or anticoagulant therapy	94 (50.5)	34 (48.6)	39 (48.1)	21 (60.0)	0.461
Antihyperlipidemic therapy	51 (27.4)	17 (24.3)	25 (30.9)	9 (25.7)	0.644
Antihypertensive therapy	131 (70.4)	50 (71.4)	54 (66.7)	27 (77.1)	0.511
TriAGe+ score	2.8 ± 0.9	3 ± 1.2	2.7 ± 0.9	2.7 ± 0.8	0.568
Low risk	85 (45.7)	31 (44.3)	37 (45.7)	17 (48.6)	0.917
High risk	101 (54.3)	39 (55.7)	44 (54.3)	18 (51.4)	
Sudbury Vertigo Risk Score	3.8 ± 3.6	4 ± 3.6	3.7 ± 4.1	3.7 ± 2.4	0.710
Low risk	109 (58.6)	41 (58.6)	51 (63.0)	17 (48.6)	0.051
Moderate risk	55 (29.6)	18 (25.7)	20 (24.7)	17 (48.6)	
High risk	22 (11.8)	11 (15.7)	10 (12.3)	1 (2.9)	

Values are presented as mean ± standard deviation, median (interquartile range), or number (percentage). Percentages in the Total column were calculated using the entire cohort as the denominator, whereas percentages in Groups A, B, and C were calculated using the number of patients within the respective admission-timing group. Percentages for normal and acute imaging findings were calculated among patients who underwent the relevant imaging modality. Group A, admission during the index ED visit; Group B, admission within 1–7 days after ED discharge; Group C, admission within 8–30 days after ED discharge. CT, computed tomography; MRI, magnetic resonance imaging; COPD, chronic obstructive pulmonary disease; TIA, transient ischemic attack; TriAGe+, Triage in Acute General Emergency score.

### 3.3 Distribution of final diagnoses

The distribution of final diagnoses among patients admitted within 30 days after presenting to the ED with vertigo is shown in Table 2. The overall distribution of the major diagnostic categories differed across the three admission-timing groups (Fisher–Freeman–Halton exact test with Monte Carlo estimation, *p* = 0.004). This comparison was exploratory, and the individual subdiagnostic categories were presented descriptively because of their small cell counts and nested structure. Neurological emergencies constituted the most frequent diagnostic category, accounting for eighty-four cases (45.2%) of all hospitalizations. Among these, stroke was the predominant diagnosis (*n* = 74, 39.8% of the entire cohort). Of the patients diagnosed with stroke, 50.0% (*n* = 37) were admitted during the index ED visit, 39.2% (*n* = 29) were admitted within 1–7 days after discharge, and 10.8% (*n* = 8) were admitted within 8–30 days after discharge. Six patients were hospitalized with other neurological disorders, while four patients were admitted because of persistent vertigo for which no specific final etiological diagnosis was established. Among the 116 admissions occurring after ED discharge, 79 (68.1%) were due to diagnoses other than stroke. Of these, 71 admissions (61.2% of all post-discharge admissions) were attributable to non-neurological conditions, whereas eight were due to other neurological diagnoses.

**Table 2:** Distribution of final diagnoses among patients admitted within 30 days of ED presentation for vertigo.

Diagnosis	Group A (*n* = 70)	Group B (*n* = 81)	Group C (*n* = 35)
Neurological emergencies (*n* = 84)	39 (55.7)	36 (44.4)	9 (25.7)
Stroke (*n* = 74)	37 (50.0)	29 (39.2)	8 (10.8)
Vertigo (*n* = 4)	0 (0)	4 (100)	0 (0)
Other neurological disorders (*n* = 6)	2 (33.3)	3 (50.0)	1 (16.7)
Internal medicine emergencies (*n* = 38)	12 (17.1)	15 (18.5)	11 (31.4)
Metabolic/renal disorders (*n* = 24)	8 (33.3)	11 (45.8)	5 (20.8)
Gastrointestinal bleeding (*n* = 10)	4 (40.0)	4 (40.0)	2 (20.0)
Other internal medicine emergencies (*n* = 4)	0 (0)	0 (0)	4 (100)
Cardiovascular emergencies (*n* = 29)	14 (20.0)	9 (11.1)	6 (17.1)
Acute coronary syndrome (*n* = 16)	7 (43.8)	4 (25.0)	5 (31.3)
Bradycardia (*n* = 8)	5 (62.5)	3 (37.5)	0 (0)
Other cardiac disorders (*n* = 5)	2 (40.0)	2 (40.0)	1 (20.0)
Infectious emergencies (*n* = 25)	4 (5.7)	17 (21.0)	4 (11.4)
Pneumonia (*n* = 11)	1 (9.1)	9 (81.8)	1 (9.1)
Cystitis (*n* = 6)	1 (16.7)	4 (66.7)	1 (16.7)
Other infectious diseases (*n* = 8)	2 (25.0)	4 (50.0)	2 (25.0)
Pulmonary emergencies (*n* = 1)	0 (0)	0 (0)	1 (2.9)
Surgical emergencies (*n* = 9)	1 (1.4)	4 (4.9)	4 (11.4)
Trauma (*n* = 6)	1 (16.7)	2 (33.3)	3 (50.0)
Other surgical diseases (*n* = 3)	0 (0)	2 (66.7)	1 (33.3)

Values are presented as number (percentage). Percentages for the major diagnostic categories were calculated within each admission-timing group and therefore represent column percentages. Percentages for the listed subdiagnoses represent the distribution of patients with that diagnosis across Groups A, B, and C and therefore represent row percentages. Group A, admission during the index ED visit; Group B, admission within 1–7 days after ED discharge; Group C, admission within 8–30 days after ED discharge. The overall distribution of the six major diagnostic categories across the three admission-timing groups was compared using the Fisher–Freeman–Halton exact test with Monte Carlo estimation based on 100,000 sampled tables (overall *p* = 0.004). Subdiagnostic categories were presented descriptively and were not subjected to separate hypothesis testing because of small cell counts and their nested structure. The overall *p* value should be interpreted as exploratory.

Internal medicine emergencies represented the second most common diagnostic group (*n* = 38, 20.4%). Within this category, acute kidney injury (*n* = 12) and sodium/glucose-related osmolarity disturbances (*n* = 12; hyponatremia, *n* = 9, and hyperglycemia-associated hyperosmolarity, *n* = 3) were the most frequent diagnoses, followed by gastrointestinal bleeding (*n* = 10). Cardiovascular emergencies accounted for 29 cases (15.6%), with acute coronary syndrome being the most common diagnosis within this group (*n* = 16), followed by bradycardia (*n* = 8). Infectious emergencies accounted for 25 admissions (13.4%), including pneumonia (*n* = 11), cystitis (*n* = 6), leptospirosis or Hantavirus infection (*n* = 2), and other infectious diseases (*n* = 6). One additional patient was admitted with another pulmonary emergency. Surgical emergencies were relatively uncommon (*n* = 9, 4.8%) and mainly consisted of trauma-related admissions. No admissions were attributable to anemia, leukemia, or another primary hematological disorder. One patient with hepatic coma was included under other internal medicine emergencies; no patient was admitted with acute liver injury.

### 3.4 Secondary analysis of stroke according to the timing of stroke-related hospital admission

As a secondary analysis, patients hospitalized with stroke were stratified according to the timing of stroke-related hospital admission: during the index ED visit, within 1–7 days after ED discharge, and within 8–30 days after ED discharge. This stratification was intended to demonstrate the heterogeneity of admission timing and should not be interpreted as indicating the onset time of stroke, diagnostic delay, or diagnostic error. The clinical characteristics of the three groups are presented in Table 3. Age and sex were comparable across the three admission-timing groups. High-priority triage was recorded in all patients admitted with stroke during the index ED visit, compared with 34.5% of patients admitted within 1–7 days and 37.5% of those admitted within 8–30 days after ED discharge (*p* < 0.001). Acute focal neurological symptoms were documented in 29.7%, 6.9%, and 0% of patients, respectively (*p* = 0.021). Cranial CT and brain diffusion MRI were performed more frequently in patients admitted during the index ED visit than in either post-discharge group (both *p* < 0.001). Gait disturbance or imbalance, syncope or presyncope, antiplatelet or anticoagulant therapy, TriAGe+ scores, and Sudbury Vertigo Risk Scores did not differ significantly across the three groups. Because only eight patients were admitted with stroke during days 8–30, these comparisons should be interpreted cautiously. All *p* values from this secondary analysis should be regarded as exploratory. In particular, comparisons involving triage acuity and neuroimaging use are closely linked to the clinical presentation and admission pathway and are therefore primarily descriptive.

**Table 3:** Clinical characteristics of patients with stroke according to the timing of stroke-related hospital admission after the index ED presentation.

Variable	Stroke-related admission during the index ED visit (*n* = 37)	Stroke-related admission within 1–7 days after ED discharge (*n* = 29)	Stroke-related admission within 8–30 days after ED discharge (*n* = 8)	*p*
Age (years)	76.1 ± 6.7	72.9 ± 6.6	74.4 ± 9.4	0.117
Male	22 (59.5)	17 (58.6)	5 (62.5)	0.981
High priority triage	37 (100)	10 (34.5)	3 (37.5)	**<0.001** ^a^ ^,^ ^b^
Acute focal neurological symptoms	11 (29.7)	2 (6.9)	0 (0)	**0**.**021**^b^
Gait disturbance/imbalance	17 (45.9)	9 (31.0)	1 (12.5)	0.151
Syncope/Presyncope	3 (8.1)	2 (5.4)	0 (0)	0.709
Cranial CT performed	37 (100)	17 (58.6)	3 (37.5)	**<0.001** ^a^ ^,^ ^b^
Brain diffusion MRI performed	37 (100)	13 (44.8)	2 (25.0)	**<0.001** ^a^ ^,^ ^b^
Antiplatelet or anticoagulant therapy	13 (35.1)	15 (51.7)	5 (50.0)	0.370
TriAGe + score	3.4 ± 1.3	2.7 ± 0.9	2.6 ± 0.5	0.065
Sudbury vertigo risk score	4.9 ± 4.1	5.2 ± 5.0	5.4 ± 1.5	0.655

Values are presented as mean ± standard deviation or number (percentage). Percentages were calculated using the number of patients within the respective stroke-related admission-timing group. Admission timing refers to the hospital admission during which stroke was recorded as the final diagnosis and does not necessarily represent the onset time of the cerebrovascular event. Continuous variables were compared using one-way analysis of variance. Overall comparisons of categorical variables were performed using the Fisher–Freeman–Halton exact test, followed, where appropriate, by pairwise Fisher's exact tests with Bonferroni correction. Superscript letters indicate statistically significant pairwise differences after Bonferroni correction.

a Index ED admission vs. admission within 1–7 days after ED discharge.

b Index ED admission vs. admission within 8–30 days after ED discharge. All *p* values should be interpreted as exploratory. CT: computed tomography; MRI: magnetic resonance imaging; TriAGe+: Triage in Acute General Emergency score.

Bold values indicate statistical significance at *p* < 0.05.

### 3.5 Selected non-neurological diagnoses after ED discharge

Selected non-neurological admissions after ED discharge were further described as cardiac (*n* = 15), metabolic (*n* = 16), and infectious (*n* = 21) conditions (Table 4). Descriptive data regarding age, sex, time to admission, triage category, intensive care unit admission, and length of hospital stay are presented in Table 4. Because of the small subgroup sizes, no formal between-group statistical comparisons were performed.

**Table 4:** Clinical characteristics of selected cardiac, metabolic, and infectious admissions after ED discharge in older adults presenting with vertigo.

Variable	Cardiac conditions (*n* = 15)	Metabolic conditions (*n* = 16)	Infectious conditions (*n* = 21)
Age (years)	77.7 ± 9.9	79.0 ± 7.0	75.1 ± 7.1
Male	9 (60.0)	9 (56.3)	12 (57.1)
Time to admission (days)	3.0 (2.0–15.0)	2.5 (1.0–11.0)	3.0 (1.0–12)
High-priority triage	1 (6.7)	4 (25.0)	6 (28.6)
Admission to intensive care unit	9 (60.0)	2 (12.5)	4 (19.0)
Length of hospital stay (days)	6.0 (3.0–10.0)	6.0 (3.0–10.8)	7.0 (4.0–12.0)

Values are presented as mean ± standard deviation, median (interquartile range), or number (percentage). Percentages were calculated within each diagnostic subgroup. Time to admission represents the number of days between the index ED visit and the subsequent unplanned acute hospital admission through an ED. Because of the small subgroup sizes, the findings are presented descriptively, and no formal between-group statistical comparisons were performed.

### 3.6 Thirty-day mortality

Among the 6,460 older adults identified through vertigo-related ICD-10 codes, 12 patients (0.19%) died within 30 days of the index ED visit. Eight deaths occurred after hospital admission among patients included in the main analytic cohort (8/186, 4.3%): four in Group A, three in Group B, and one in Group C. Four additional patients died without any recorded hospital admission during the follow-up period, corresponding to 0.06% of the screened population. These deaths occurred on days 4, 7, 15, and 26 after the index ED visit.

## 4 Discussion

In this province-wide cohort of adults aged 65 years and older presenting to the ED with vertigo, 186 patients with vertigo confirmed as the dominant presenting complaint were included in the final 30-day hospital admission cohort. The main contribution of this study is the descriptive characterization of diagnoses among older adults who were hospitalized within 30 days after an ED presentation with vertigo. Among patients admitted after ED discharge, non-neurological conditions constituted a substantial proportion of the diagnostic spectrum. Stroke remained the most frequent single diagnosis; however, renal/metabolic, infectious, cardiac, pulmonary, and surgical conditions were also represented. Because the main analytic cohort was restricted to patients who were subsequently hospitalized, these findings cannot be used to estimate the overall risk of admission after vertigo, assess the diagnostic accuracy of the index ED evaluation, or determine whether a later diagnosis was missed during the initial visit.

The absence of specific peripheral vestibular diagnoses, such as benign paroxysmal positional vertigo, persistent postural-perceptual dizziness, vestibular neuritis, or Ménière's disease, should be interpreted in light of the study design. The analytic cohort was restricted to patients who underwent an unplanned acute hospital admission through an ED within 30 days after the index visit. Patients managed without hospitalization or admitted electively through outpatient pathways were outside the scope of the study. Therefore, the absence of these diagnoses from the final cohort does not imply that peripheral vestibular disorders were absent from the broader ICD-code–screened population. Four patients were admitted because of persistent vertigo, but no specific final etiological diagnosis was established during hospitalization.

The association between acute dizziness and stroke has been extensively examined in the emergency medicine literature. Among patients presenting to the ED with acute dizziness or vertigo, the reported incidence of stroke is generally low but clinically meaningful, most often ranging between 2% and 5% (1, 2). In patients with acute vestibular syndrome, the proportion of central causes may be higher, and posterior circulation stroke remains a diagnostic concern (4). Furthermore, studies have shown that although the short-term risk of stroke following ED discharge for dizziness is low, it is not negligible and may still be associated with important functional outcomes (3). Accordingly, stroke remains an essential concern in the evaluation of ED vertigo, but it does not fully explain the broader spectrum of serious diagnoses in older adults.

Unlike most previous studies, which have focused primarily on stroke incidence, the present study described the broader diagnostic spectrum of acute hospital admissions occurring within 30 days after the index ED presentation. However, temporal association alone does not establish that a later diagnosis was already present or unrecognized during the initial evaluation.

The relationship between the index vertigo presentation and subsequent hospitalization is particularly uncertain for admissions occurring later in the 30-day follow-up period. Older adults have a substantial background risk of hospital admission because of multimorbidity and age-related vulnerability, and some conditions diagnosed 8–30 days later may have developed independently of the index presentation (12). This possibility is especially relevant for diagnoses such as trauma and pneumonia, which may represent new clinical events rather than manifestations of the original vertigo episode. Accordingly, the diagnoses observed during follow-up should be interpreted as temporally associated outcomes within the predefined observation period, rather than as conditions necessarily caused by or present at the index ED visit.

This uncertainty was also evident in the stroke subgroup. Of the 37 post-discharge stroke-related admissions, 29 occurred within 1–7 days and eight occurred within 8–30 days. Although stroke-related admission shortly after discharge may be more plausibly associated with the index symptom episode, the present retrospective data cannot determine whether the cerebrovascular event was already present, evolving, or unrecognized during the initial ED evaluation. This uncertainty is greater for admissions occurring during days 8–30, which may represent newly developed cerebrovascular events. Therefore, the timing of stroke-related admission should be interpreted descriptively and should not be considered a direct indicator of stroke onset, diagnostic delay, or diagnostic error.

Non-neurological diagnoses identified after ED discharge included acute kidney injury, sodium/glucose-related osmolarity disturbances, infections, and cardiac conditions. Previous studies have shown that hyponatremia and acute kidney injury may present with nonspecific symptoms in emergency populations (13–16), while bradycardia and drug-related conduction disturbances are important causes of hospitalization in older adults (17, 20, 21). Infectious diseases may also present atypically in this age group (18, 19), and older patients with nonspecific complaints have high short-term admission and mortality rates (12). These observations provide clinical context for the diagnostic spectrum identified in our cohort; however, the present data do not establish that these conditions caused the index vertigo or were already present during the initial ED evaluation. Medication-related dizziness and polypharmacy should also be considered in older adults, in whom multimorbidity frequently results in exposure to multiple medications with potential vestibular, cardiovascular, or central nervous system adverse effects (22). Medication burden, particularly exposure to anticholinergic and sedative drugs, has been associated with dizziness, balance impairment, and poorer functional outcomes in older patients (23).

Clinical risk scores such as TriAGe+ and the Sudbury Vertigo Risk Score were developed to estimate the probability of serious underlying causes in patients presenting to the ED with vertigo (8, 9). External validation studies have demonstrated moderate diagnostic performance, particularly for identifying central neurological pathology (10, 11). However, advanced age and a higher burden of comorbidity may alter clinical presentation and influence the discriminatory performance of such risk scores (9, 11). In older adults, atypical symptom patterns and the higher prevalence of non-specific complaints may limit the applicability of standard risk stratification tools (12). Because these scores were originally developed primarily to identify central neurological causes of dizziness, their ability to capture serious non-neurological systemic conditions in older adults may be more limited.

In our cohort of patients aged ≥65 years, risk scores did not clearly distinguish between those admitted during the index visit and those hospitalized later within 30 days. This finding may be related to the substantial proportion of serious non-neurological conditions identified in our population. This is particularly relevant when the principal diagnostic concern at the index visit is stroke, whereas the subsequent admission may ultimately reflect a systemic rather than neurological cause. At the same time, because our study included only patients in whom vertigo was the predominant presenting symptom, and because cases with more overt systemic features may have followed different clinical pathways, this observation should not be interpreted as a definitive judgment on the overall performance of these risk scores. Rather, our results suggest that, in older adults, vertigo should be assessed within a broader clinical context that acknowledges the heterogeneous nature of serious underlying disease. Although complete documentation was available for all required score components, both scores were calculated retrospectively from routine clinical records rather than being applied prospectively at the bedside. Therefore, the present findings should be regarded as exploratory and should not be interpreted as a formal validation of either score.

The evaluation of vertigo in the ED is often conducted within a diagnostic framework primarily aimed at the rapid exclusion of central pathology. Structured bedside examination strategies and related algorithms have shown high sensitivity for central causes when applied by appropriately trained clinicians (6, 7). In addition, conceptual approaches based on timing and triggers have been proposed to facilitate the clinical differentiation of vestibular syndromes (5). Nevertheless, the optimal use of consultation and imaging strategies in ED vertigo management remains debated (24). Within this framework, conditions that do not initially present with overt neurological features may receive less diagnostic emphasis at the index visit.

Patients admitted during the index ED visit more frequently had high-priority triage, acute focal neurological symptoms, and neuroimaging than patients admitted after ED discharge. These findings are descriptive and likely reflect differences in presenting clinical features and subsequent care pathways. Because the study did not include a comparison group of patients who were never hospitalized and did not use prospective diagnostic adjudication, these observations should not be interpreted as evidence of the accuracy or effectiveness of triage and imaging strategies, or as proof that diagnoses were missed during the index ED visit. Furthermore, given the number of comparisons performed and the absence of a global multiplicity adjustment, the associated *p* values should be interpreted as exploratory rather than confirmatory. The study also cannot determine whether broader testing, observation, or follow-up strategies would have altered subsequent admission or clinical outcomes.

### 4.1 Strengths and limitations

The principal strength of this study lies in its province-wide dataset and systematic 30-day follow-up of older adults presenting to the ED with vertigo. Inclusion of all eligible patients across seventeen hospitals over a two-year period provided a real-world cohort reflecting routine emergency practice. In contrast to many previous studies focused primarily on stroke outcomes, we examined the broader diagnostic spectrum of hospital admissions after vertigo presentation. Another important strength is that case identification was not based solely on ICD coding; electronic records were reviewed to confirm that vertigo was the dominant presenting complaint.

Several limitations should be acknowledged. First, the retrospective design precludes causal inference and depends on the completeness of clinical documentation. Second, because the main analytic cohort included only patients with a recorded hospital admission within 30 days, the study cannot estimate the overall probability of admission after an ED presentation with vertigo, evaluate the diagnostic accuracy of the index ED assessment, or determine whether a subsequent diagnosis was missed during the initial visit. Similarly, the recorded timing of stroke-related admission does not establish the onset time of the cerebrovascular event; particularly for strokes admitted during days 8–30, a newly occurring event cannot be distinguished from a condition related to the index presentation. In addition, the study cannot determine whether hospitalizations occurring later in the follow-up period were clinically related to the index vertigo presentation; some admissions, particularly those due to trauma, pneumonia, or other newly developed conditions, may have been independent events. Although 30-day mortality was assessed for the entire ICD-code–screened population using the national Death Notification System, the causes of death were not adjudicated; therefore, the clinical relationship between these deaths and the index ED presentation could not be established. Third, although electronic records were reviewed to confirm that vertigo was the dominant presenting complaint, some degree of symptom-classification uncertainty is unavoidable in routine ED documentation. Case identification was restricted to four vertigo-related ICD-10 codes. Although these codes represented the routinely used coding pathway for vertigo-related ED presentations in the provincial hospital information system, some older adults presenting with dizziness or vertigo may have been coded under another symptom or final diagnosis and therefore may not have been captured. Fourth, hospital admissions occurring outside the provincial network may not have been captured, and local triage practices, admission thresholds, and referral patterns may limit generalizability to other settings. Finally, although all required components of the TriAGe+ and Sudbury Vertigo Risk Score were completely documented for every patient, the scores were reconstructed retrospectively from routine clinical records rather than administered prospectively using a standardized study protocol. Complete longitudinal medication histories were not consistently available; therefore, polypharmacy and medication-related dizziness could not be evaluated systematically in the present cohort. Accordingly, the score comparisons should be considered exploratory and should not be interpreted as prospective validation of these instruments. Nevertheless, the study reflects routine ED practice and highlights an underexplored systemic dimension of vertigo evaluation in older adults.

## 5 Conclusions

Among older adults admitted within 30 days after an ED presentation with vertigo, non-neurological conditions accounted for a substantial proportion of hospitalizations after ED discharge. Stroke remained the most frequent single diagnosis, but renal/metabolic, infectious, cardiac, pulmonary, and surgical diagnoses were also observed. These findings are descriptive and apply only to patients who were subsequently hospitalized; they do not estimate the overall risk of hospital admission, the diagnostic accuracy of the index ED evaluation, or whether later diagnoses were missed during the initial visit.

## Data Availability

The raw data supporting the conclusions of this article will be made available by the authors, without undue reservation.
